# Physical health mindsets and information avoidance

**DOI:** 10.1007/s10865-024-00514-1

**Published:** 2024-09-21

**Authors:** Abigail G. O’Brien, Jeremy L. Foust, Jennifer M. Taber

**Affiliations:** Department of Psychological Sciences, Kent State University, 390 Kent Hall, Kent, OH 44242-0001 USA

**Keywords:** Information avoidance, Risk calculator, Growth and fixed mindsets, Incremental and entity implicit theories, Health, Risk

## Abstract

Health mindsets refer to beliefs about the malleability (growth mindset) versus stability (fixed mindset) of physical health and have gained traction as a predictor of health beliefs and behaviors. Across two studies, we tested whether health mindsets were associated with avoiding personalized health risk information. In Study 2, we also tested whether conceptually-related constructs of internal and chance health locus of control, health self-efficacy, fatalism, and genetic determinism were associated with information avoidance. Health mindsets were manipulated in Study 1 (college students, *n* = 284; 79.58% female; *M*_age _= 19.74) and measured in Study 2 (participants recruited through MTurk, *n* = 735; 42.04% female; *M*_age _= 35.78). In both studies, participants viewed a prediabetes infographic and were informed they could learn their prediabetes risk by completing an online risk calculator. Behavioral obligation was also manipulated in both studies to test whether an additional behavioral requirement associated with learning one’s risk would exacerbate any negative impact of health mindsets on avoidance rates. All participants then indicated their interest in learning their prediabetes risk (avoidance intentions) and decided whether to complete the online risk calculator (avoidance behavior). In Study 1, there was no impact of health mindsets, behavioral obligation, or their interaction on avoidance intentions or behavior. Study 2 similarly did not provide consistent evidence for an association of health mindsets, behavioral obligation, or their interaction with avoidance intentions or behavior. However, in Study 2, internal health locus of control was consistently associated with both intentions and behavior. Health information avoidance may be a barrier to prevention and early detection of disease. To encourage individuals to learn potentially important health information, public health interventions might seek to increase people’s beliefs that their own actions play a role in their health outcomes. Interventions may also seek to increase people’s knowledge about and skills regarding improving their health outcomes, which may influence health locus of control beliefs.

**Supplementary Information:**

The online version contains supplementary material available at 10.1007/s10865-024-00514-1.

Mindsets—also known as implicit theories—refer to people’s beliefs about the stability and changeability of personal attributes (Dweck et al., 1995a). People with stronger *fixed* mindsets believe attributes are more stable and unchangeable. People with stronger *growth* mindsets believe attributes, through effort, can change and improve. Originally developed in the domain of academic achievement (Dweck et al., 1995a) and assessed with respect to intelligence (Blackwell et al., 2007; Yeager et al., 2019), mindsets are also commonly assessed about personality (Chiu et al., 1997; Miu & Yeager, 2015). Mindsets about aspects of one’s health—including weight (Burnette, 2010; Ehrlinger et al., 2017; Lyons et al., 2015), smoking (Thai et al., 2018, 2020), fitness and athletic ability (Orvidas et al., 2018), and general health (Bunda & Busseri, 2019; John-Henderson et al., 2021; Thomas et al., 2019)—have gained traction as correlates and predictors of health beliefs and behaviors. The present studies built on research suggesting that growth mindsets might counter defensive responses to threatening information (Taber et al., 2017). We tested the hypothesis that individuals with stronger fixed versus growth mindsets about physical health would be more likely to avoid potentially threatening health information and that a behavioral obligation associated with learning the information would moderate this association. A secondary aim was to examine the relationship between health mindsets and other conceptually similar agency-related beliefs.

Overall, people with stronger growth mindsets in the domains of health, intelligence, and personality tend to engage in more adaptive, approach-oriented behaviors than people with stronger fixed mindsets (Dweck & Leggett, 1988). Within a health context, individuals with stronger growth mindsets are more likely to report engaging in physical activity and eating healthier foods (Ehrlinger et al., 2017; Lyons et al., 2015; Orvidas et al., 2018; Parent & Alquist, 2016); they are also less likely to use avoidant behaviors during a potentially threatening situation (e.g., failing to reach a dieting goal or considering whether to learn genomic sequencing results; Burnette, 2010; Kasimatis et al., 1996; Taber et al., 2017). Individuals with fixed mindsets may view remedial action to improve as unnecessary and even threatening—that is, it would simply prove their shortcomings (Hong et al., 1999). When faced with a threat, or a potential challenge to one’s self-integrity and adequacy (Steele, 1988), those with a fixed mindset might be more likely to respond defensively to cope with negative affect elicited from a potential threat (McQueen et al., 2013). Information avoidance—behaving in a way that prevents or delays the receipt of available but possibly unwanted information (Sweeny et al., 2010)—is a specific behavior that can indicate defensiveness (McQueen et al., 2013). People may defensively avoid available information, such as whether they are at greater risk for a disease (Orom et al., 2021), if they believe learning the information will make them feel bad (Foust & Taber, 2023). People are also more likely to avoid information that may demand some undesired behavioral action such as additional medical exams (Howell & Shepperd, 2013). Further, control-related beliefs such as fatalism (Emanuel et al., 2015; Orom et al., 2021), locus of control (Orom et al., 2021), and self-efficacy (Hua & Howell, 2022) are associated with information avoidance. In the present research, we built on this prior evidence by examining the relationship between avoidance and another control-related individual difference: mindsets.

Prior research suggests that health mindsets may be associated with conceptually similar constructs that assess beliefs about the controllability of health and disease, including locus of control beliefs (Schreiber et al., 2020) and beliefs about the role of genetics (John-Henderson et al., 2021). We aimed to add to this preliminary evidence by assessing health locus of control (Wallston et al.,1978), health self-efficacy (Becker et al.,1993), fatalism (Shen et al., 2009), and genetic determinism (Parrott et al., 2012) to determine their associations with health mindsets and with health information avoidance. As each of these constructs relates to individuals’ beliefs about factors that contribute to or determine their health—and especially beliefs about one’s agency in controlling future health outcomes—we collectively refer to health mindsets, health locus of control, health self-efficacy, fatalism, and genetic determinism, as *agency beliefs* (a term used by Orom et al., 2021 to describe a set of similar measures). Identifying which of multiple possible agency beliefs (if any) is most strongly associated with information avoidance can provide guidance for future research as to what constructs to assess. The results of these analyses may also point to specific factors that interventions to decrease information avoidance may target, thus informing the work of public health scientists and officials designing interventions or public health messaging to promote engagement with health information.

Finally, although previous research has suggested that people can be a growth theorist in one domain and a fixed theorist in another (Dweck et al., 1995a; Molden & Dweck, 2006), to our knowledge, general physical health mindsets have not been directly compared to mindsets in other domains. As such, we also examined the association of health mindsets with weight and intelligence mindsets to determine the uniqueness of health mindsets.

## Current studies and hypotheses

We tested two broad aims across two studies. The primary aim was to test the relationship between health mindsets and information avoidance. We hypothesized that participants with a weaker versus stronger growth mindset would be more likely to avoid learning personalized health risk information. We also tested the impact of behavioral obligation on avoidance. People are more likely to avoid available information when that information may require an undesired behavior (Sweeny et al., 2010), such as needing to undergo an unpleasant medical procedure after learning one’s disease risk (Howell & Shepperd, 2013). Thus, we hypothesized a main effect that would replicate prior research, such that participants who were informed of a behavioral requirement if they learned they were at high risk (high behavioral obligation message) would be more likely to avoid learning their risk than participants who were not informed of a behavioral requirement (low behavioral obligation message). Further, we hypothesized that health mindsets would interact with behavioral obligation such that those with a weaker growth mindset who were informed of an additional behavioral obligation prior to making their decision would be most likely to avoid their risk. We predicted this interactive effect because we anticipated that a fixed mindset would be more detrimental when participants faced an additional barrier to learning information.

The secondary aim was to investigate the extent to which health mindsets were associated with other health agency beliefs (i.e., health locus of control, health self-efficacy, fatalism, and genetic determinism) and to examine the associations among health mindsets, weight mindsets, and intelligence mindsets. We hypothesized that health mindsets would be significantly associated with health locus of control, health self-efficacy, fatalism, genetic determinism, and weight mindsets but that health mindsets would not be significantly associated with intelligence mindsets. Finally, we hypothesized that individuals with lower internal health locus of control, greater chance health locus of control, lower self-efficacy, greater fatalism beliefs, and greater genetic determinism beliefs would be more likely to avoid health information.

## General method

We conducted two studies with slightly different methodology in the Fall of 2019. Both studies were approved by Kent State University’s Institutional Review Board and all respondents provided informed consent. In Study 1, college students visited a laboratory and were randomly assigned to a growth versus a fixed health mindset induction. In Study 2, adult participants recruited online through Amazon’s MTurk completed a battery of individual difference measures, including mindsets and health agency beliefs. Surveys were administered through Qualtrics. In both studies, participants were given the opportunity to complete an online risk calculator to learn their risk of prediabetes. Prior to deciding whether to complete the risk calculator, participants in both studies were randomly assigned to 1 of 2 behavioral obligation conditions. Those who were willing to learn their risk for prediabetes completed a prediabetes risk calculator.

Prediabetes was chosen for several reasons. First, individuals with prediabetes typically do not experience symptoms (Centers for Disease Control and Prevention [CDC], 2022); determining whether someone is at risk requires a healthcare provider checking one’s HbA1c levels or calculating one’s risk through an online prediabetes risk calculator. Second, a prediabetes diagnosis is reversible: individuals can decrease their blood sugar levels through behaviors such as exercise (CDC, 2022). Thus, if a person avoids learning their risk they may miss the opportunity to act before prediabetes develops into type 2 diabetes. Further, prediabetes is relatively common and most people who have it are unaware that they do (CDC, 2022).

The study design, hypotheses, and analytic plan were pre-registered on Open Science Framework (OSF; Study 1: bit.ly/Study1mindsets, Study 2: bit.ly/Study2Mindsetsoriginal and bit.ly/Study2Mindsetsupdated). Minor deviations from the pre-registered analytic plan are described throughout the text and in footnotes. In the interest of brevity, the methods and results of some pre-registered analyses deemed not central to the present study are reported in Electronic Supplementary Materials (ESM) Section I. Another manuscript using data from these datasets examines open-ended reasons for avoiding one’s prediabetes risk information only among the subset of respondents who avoided (O’Brien et al., 2024).

## Study 1

### Participants

Participants were recruited through Kent State University’s Department of Psychological Science’s subject pool. Eligibility criteria included aged 18 years or older and no self-reported history of prediabetes, type 1 diabetes, or type 2 diabetes. Participants were recruited in two ways: (1) to complete the experiment in person in a psychology lab (*n* = 244), and (2) to serve as a control group for the mindset manipulation by completing an online survey (*n* = 100). The control group did not receive either a mindset or a behavioral obligation manipulation and the data were used simply to determine the average of physical health mindsets in the absence of any mindset manipulation. Control participants completed the study online to save time and labor. We hereafter refer to these participants as the “lab experiment” and “control” participants, respectively. Following exclusions (see ESM Section II for details), the analytic sample size was 197 lab participants and 87 control participants. A post hoc power analysis conducted in G*Power (Faul et al., 2009) indicated that a sample of 197 respondents would allow us to use a 2 by 2 ANOVA to detect a medium-small effect size of 0.204 (consistent with the effect size in Bunda and Busseri’s (2019) manipulation of mindsets) with power of 0.81.^1^

### Design and procedure

The study used a 2 (mindset: growth versus fixed) by 2 (behavioral obligation: high versus low) fully-crossed experimental design. In the lab experiment, participants were first given the mindset manipulation, depending on their randomly assigned condition. All participants were then shown an infographic (see ESM Section III) containing accurate information about prediabetes compiled from the CDC and the American Diabetes Association and citing these sources. The infographic was designed to emphasize the negative health consequences of prediabetes, as well as its relatively high prevalence, and did not focus on preventability. Our aims for this were twofold. First, by focusing on the negative consequences of prediabetes, individuals should feel more threatened and therefore be more likely to avoid learning their risk (Taber et al., 2015). Second, emphasizing the prevalence was intended to increase the perceived relevance of prediabetes, thereby increasing threat and motivation to avoid learning personal risk information (Jemmott et al., 1986). After viewing the infographic, all participants read that they could learn their risk of prediabetes: “The American Diabetes Association has developed an accurate way to calculate your risk of prediabetes. You can learn your risk by answering several questions about yourself. If you score higher than a certain number, you are likely to have prediabetes and are at high risk of type 2 diabetes.” Participants were then shown the behavioral obligation manipulation. Next, they reported their intentions to learn their risk and either opted to learn or avoid their risk. Participants who indicated they would like to learn their risk were redirected to an external CDC website (CDC, n.d.). Upon returning to the Qualtrics survey, they were asked to report their results and continue with the survey. Regardless of whether participants learned their risk, they then completed the items in the prediabetes risk calculator as part of the Qualtrics survey. This allowed us to manually calculate participants’ prediabetes risk by summing the number of risk factors (see ESM Section IV). Participants then completed demographic questions and other measures not discussed in the current study. Finally, participants were debriefed and given resources.

The control participants completed a health mindset scale, were exposed to the prediabetes infographic, reported their intentions to learn their risk, and then either completed the prediabetes risk calculator or avoided. No information was provided with respect to the behavioral obligation manipulation.

### Mindset manipulation

Participants were randomly assigned to 1 of 2 mindset manipulation conditions: growth (coded as 1 for analyses) or fixed (coded as 0 for analyses). In both conditions, participants were presented with a physical copy of a Psychology Today-type article adapted slightly (only one sentence was changed) from Bunda and Busseri (2019; see ESM Section V for the full text). In the growth mindset condition, participants read information supporting the malleability of a person’s physical health, emphasizing that health can change. In the fixed mindset condition, participants read information supporting the stability of a person’s health, suggesting health does not change. Consistent with Bunda and Busseri (2019), after reading the article participants were asked two open-ended questions to encourage deeper-level processing and comprehension (e.g., “Please briefly describe how this article relates to your health, and to your life. Provide 2–3 examples from your personal experience.”). To check comprehension, participants were asked, “In the article you just read, Dr. Gregory Pierce’s research findings suggests that our health is what?” with response options including, “determined by people’s behavior,” “determined by people’s genetics,” or “don’t know.” Participants in the growth mindset condition were excluded from analyses if they answered anything other than “determined by people’s behavior.” Participants in the fixed mindset condition were excluded from analyses if they answered anything other than “determined by people’s genetics.”

### Behavioral obligation manipulation

After exposure to the prediabetes infographic, and prior to deciding whether to learn their risk, participants in the lab experiment were randomly assigned to 1 of 2 behavioral obligation conditions: high (coded as 1 for analyses) or low (coded as 0 for analyses). Participants in the high behavioral obligation condition were informed that if they learned their risk and their risk was high, they would be contacted by a physician from the health center on campus to schedule blood work for a definitive diagnosis. Participants in the low behavioral obligation were informed that if they learned their risk, and their risk was high, they would be given information about what to do next. See ESM Section VI for the exact wording of each condition.

### Measures

Information avoidance was assessed as intentions and behavior. *Avoidance intentions* was assessed with: “How interested are you in learning your risk of prediabetes?” on a scale from 1 (*Not at all interested*) to 5 (*Extremely interested*), reverse scored so higher scores indicated greater avoidance intentions. *Avoidance behavior* was a composite of two items. When deciding whether to avoid, participants were asked, “Would you like to calculate your risk today?” with response options including “yes” or “no.” Those who answered “yes” to this initial question were then asked, “Based on the results of the test, what was your score?” Response options for this second question included, “I did not learn my risk.” Participants were considered to have avoided if they responded “no” to the first question or if they responded “yes” to the first question but responded “I did not learn my risk” to the second question.

*Health mindsets* (Bunda & Busseri, 2019) were assessed as the average of six items (e.g., “I can’t change whether I’m in good health or poor health”; *α* = 0.880) from the implicit theory of health scale measured on a scale from 1 (*strongly agree*) to 6 (*strongly disagree*). This measure was used as a manipulation check for the mindset manipulation. Items were reverse scored as necessary so that higher scores indicate stronger growth mindsets.

One item served as a *behavioral obligation manipulation check.* Immediately following exposure to either the high or low behavioral obligation message, participants in the lab experiment were asked the following question, “According to this information, if you learn that you are at high risk of prediabetes, what should you do?” Response options included, “Wait to be contacted by a physician,” “I have not been given this information yet,” and “I don’t know.” Participants in the high behavioral obligation condition were coded as responding correctly if they selected, “Wait to be contacted by a physician” and as responding incorrectly if they selected a different response. Participants in the low behavioral obligation condition were coded as responding correctly if they selected, “I have not been given this information yet” and as responding incorrectly if they selected a different response.

Participants also reported demographic characteristics, including age, gender, and race and ethnicity (see Table 1).

**Table 1:** Sociodemographic characteristics of participants

	Study 1, *n* = 284		Study 2, *n* = 735	
	*n*	%	*n*	%
Gender ^a^				
Male	58	20.42	426	57.96
Female	226	79.58	309	42.04
Other not listed	0	0	–	–
Prefer not to say	0	0	–	–
Race and Ethnicity ^b^				
Asian	6	2.11	52	7.07
Black or African American	39	13.73	97	13.20
Native Hawaiian or Pacific Islander	1	0.35	2	0.27
American Indian or Alaska Native	2	0.70	12	1.63
White	239	84.15	577	78.50
Latino, Hispanic, or Mexican American	14	4.93	66	8.98
Other	9	3.17	20	2.72
Would rather not report	3	1.06	–	–
Education				
Less than 8 years	–	–	0	0
9 through 11 years	–	–	1	0.14
12 years or completed high school	–	–	96	13.06
Post high school training other than college (vocational or technical)	–	–	16	2.18
Some college	–	–	200	27.21
College graduate	–	–	348	47.35
Postgraduate	–	–	74	10.07
	*M*	*SD*	*M*	*SD*
Age in years	19.74	1.93	35.78	10.06
Objective prediabetes risk ^c^	1.11	1.09	2.40	1.61

^a^ Gender was asked using a binary question format in Study 2

^b^ Categories are not mutually exclusive

^c^ Participants could receive a value ranging from 0–10

### Overview of analyses

We first examined the effectiveness of the mindset manipulation by testing whether mindsets differed across the growth, fixed, and control conditions. We also examined the proportion of correct responses to the behavioral obligation manipulation check. Consistent with recommendations (Mutz et al., 2019), we did not test whether any factors differed across experimental conditions or consider including any covariates in analyses. We then tested the interactive effects of growth and fixed mindsets and high and low behavioral obligation on avoidance intentions and behavior using 2 by 2 ANOVAs and logistic regressions. Because of a high rate of incorrect responses to the behavioral obligation manipulation check, analyses involving behavioral obligation were conducted in two ways: based on randomly assigned condition and based on how participants responded to the manipulation check question.^2^

### Study 1 results

#### Preliminary analyses

Table 1 provides demographic characteristics of the sample. On average, participants’ risk for prediabetes was low, *M* = 1.11 on a scale from 0 to 10, *SD* = 1.09. Participants’ intentions to avoid their prediabetes risk was about at the midpoints of the scale, *M* = 2.79 out of 5, *SD* = 1.09, and 29.23% (*n* = 83) of participants avoided learning their risk of prediabetes.

To determine the effectiveness of the mindset manipulation, a one-way ANOVA was run to examine mean differences across the growth, fixed, and control conditions. Mindsets differed significantly across conditions, *F*(2, 253.15) = 43.87, *p* < .001, η² = 0.24: individuals in the growth mindset condition reported significantly stronger growth mindsets, *M* = 5.20 out of 6, *SD* = 0.60, than individuals in the control, *M* = 4.73, *SD* = 0.78, *p* < .001, and fixed conditions, *M* = 4.18, *SD* = 0.89, *p* < .001. Likewise, individuals in the control condition reported significantly stronger growth mindsets than individuals in the fixed condition, *p* < .001. Thus, the articles successfully altered health mindsets in the manner intended. Of note, participants in all three conditions reported mindsets significantly above the midpoint of the scale, all *p*s < 0.001. Thus, the growth manipulation strengthened growth mindsets as intended, but the fixed mindset manipulation merely weakened growth mindsets, rather than creating fixed mindsets.

In the high behavioral obligation condition, 77.00% (77 of 100) of participants correctly answered the manipulation check, and 88.54% (85 of 96) of participants in the low behavioral obligation condition correctly answered the manipulation check (one participant did not answer this question). While we originally preregistered excluding participants who answered incorrectly, given the number of participants who answered incorrectly or did not answer (*n* = 35), we retained all participants in analyses.

#### Information avoidance intentions and behavior

We conducted a 2 (growth vs. fixed mindset) by 2 (high vs. low behavioral obligation) ANOVA to test for main effects and the interaction of the health mindset manipulation and behavioral obligation manipulation on avoidance intentions. Inconsistent with hypotheses, there was no main effect of mindset condition, *F*(1, 193) = 0.05, *p* = .825, η_*p*_² < 0.001, or behavioral obligation condition, *F*(1, 193) = 0.12, *p* = .727, η_*p*_² = 0.001. Likewise, our hypothesis that participants in the fixed mindset condition who read the high behavioral obligation message would report the strongest intentions to avoid was not supported, interaction effect: *F*(1, 193) = 0.05, *p* = .821, η_*p*_² < 0.001. When analyses were rerun based on how participants responded to the behavioral obligation manipulation check rather than their randomly assigned condition, all effects remained nonsignificant.

Logistic regression was used to test for main effects and the interaction of the health mindset and behavioral obligation manipulations on avoidance behavior. Inconsistent with hypotheses, there was no main effect of mindset condition, *OR* = 0.75, *p* = .551, 95% CI [0.29, 1.94], or behavioral obligation condition, *OR* = 1.29, *p* = .580, 95% CI [0.53, 3.15]. The interaction effect was also nonsignificant, *OR* = 1.35, *p* = .647, 95% CI [0.38, 4.83]. When analyses were rerun based on how participants responded to the behavioral obligation manipulation check rather than randomly assigned condition, all effects remained nonsignificant.

### Study 1 discussion

The aim of Study 1 was to experimentally test the effect of health mindsets (and behavioral obligation) on information avoidance. Despite successfully manipulating health mindsets, health mindsets did not appear to impact avoidance intentions or behavior. Similarly, informing participants they would need to follow up with a provider to get additional testing (i.e., high behavioral obligation) did not increase avoidance intentions or behavior. One explanation for the null findings is that the young adult sample did not perceive prediabetes as threatening or relevant enough to find an effect of mindsets on avoidance. The specific disease of prediabetes may be more threatening to a general adult population.

## Study 2

In Study 2, we tested associations among participants’ naturally occurring health mindsets and avoidance intentions and behavior in a general adult sample. Similar to Study 1, we also tested whether there was an interactive effect of health mindsets and behavioral obligation on health information avoidance. We built on Study 1 by also examining the association of health mindsets with weight and intelligence mindsets as well as with five conceptually similar health agency constructs. Finally, we tested whether these other health agency beliefs were associated with avoidance intentions and behavior.

### Participants

Participants were recruited using Amazon’s Mechanical Turk (MTurk), a crowdsourcing platform. Data using MTurk has been shown to be of similar quality to in-person data collection (Casler et al., 2013). We used TurkPrime’s MTurk Toolkit (Litman et al., 2017) to reduce the number of low-quality responses by excluding workers from suspicious geolocations (Gautam, 2018). In line with suggestions to use response validity indicators to screen out potentially bad data (Chmielewski & Kucker, 2020), we collected and used qualitative data to determine the quality of responses. In total, we collected data from 1039 individuals and excluded 288 participants from analyses (see ESM Section VII for details), resulting in a final analytic sample size of 735. A post hoc power analysis indicated that the recruited sample of 735 allowed us to detect a small effect size of 0.02 at *p* < .05 power using a linear regression with 7 predictors.^3^

### Design and procedure

Eligibility criteria included aged 18 years or older, currently living in the U.S., and no self-reported history of prediabetes, type 1 diabetes, or type 2 diabetes. The procedure was the same as Study 1, except mindsets were measured rather than manipulated. Participants first completed the following measures in a randomized order: health locus of control, health self-efficacy, fatalism, and genetic determinism. Then, participants completed the health, intelligence, and weight mindset measures in a randomized order. Next, participants were shown the prediabetes infographic described in Study 1. Participants were told they could learn their prediabetes risk by completing the risk calculator, viewed the high or low behavioral obligation manipulation depending on randomly assigned condition, reported their intentions to avoid, and either learned their risk or avoided. Regardless of whether participants learned their risk or avoided, they completed the items in the prediabetes risk calculator as part of the Qualtrics survey to allow us to manually calculate prediabetes risk. Participants then completed demographic questions and other measures not discussed in the current study. Finally, participants were debriefed and given resources.

### Behavioral obligation manipulation

Participants read the same message described in Study 1 regarding the opportunity to learn their prediabetes risk by completing the online risk calculator. Participants assigned to the high behavioral obligation condition (coded as 1 for analyses) read that adults at high risk of prediabetes should make an appointment with a physician to receive additional testing for a definitive diagnosis. Those in the low behavioral obligation condition (coded as 0 for analyses) read that if they learned they were at high risk of prediabetes they would be given information about what to do next. Exact wording of these conditions can be found in ESM Section VIII.

### Measures

The measures used to assess participants’ health mindsets, information avoidance intentions, and information avoidance behavior were the same as described in Study 1. Measures unique to Study 2 are described below.

*Internal health locus of control* (Wallston et al., 1978) was assessed as the average of six items (e.g., “I am in control of my health;” *α* = 0.788) on a scale from 1 (*strongly disagree*) to 6 (*strongly agree*). Higher scores indicate greater beliefs that one’s behavior controls one’s health. *Chance health locus of control*^4^ (Wallston et al., 1978) was assessed as the average of six items (e.g., “My good health is largely a matter of good fortune;” *α* = 0.800) on a scale from 1 (*strongly disagree*) to 6 (*strongly agree*). Higher scores indicate greater beliefs that chance or luck determines one’s health. *Health self-efficacy* (Becker et al., 1993) consists of four subscales on a scale from 0 (*Not at all*) to 4 (*Completely*): nutrition (six items), well-being (seven items), exercise (seven items), and responsible health practices (eight items). Participants were asked to, “please rate how well you are able to perform each of the following health practices.” We measured health self-efficacy as the average of all 21 items that comprise the exercise (e.g., “Do exercises that are good for me”), nutrition (e.g., “Eat a balanced diet”), and healthy practices (e.g., “Watch for negative changes in my body’s condition”) subscales (*α* = 0.923). We determined these subscales to be the most relevant to prediabetes prevention because they include behaviors that can lower prediabetes risk. *Fatalism* (Shen et al., 2009) was assessed as the average of 20 items (e.g., “If someone is meant to get a serious disease, they will get that disease”; *α* = 0.935) on a scale from 1 (*strongly disagree*) to 5 (*strongly agree*). Higher scores indicate greater beliefs that one’s health is predetermined and determined by luck. *Genetic determinism* (Parrott et al., 2012) was assessed as the average of 14 items (e.g., “Genes are more important than one’s own behavior in determining one’s health”; *α* = 0.879) on a scale from 1 (*strongly disagree*) to 5 (*strongly agree*). Higher scores indicate greater beliefs that a person’s health is determined by their genes.

*Weight mindsets* (Burnette, 2010) were assessed as the average of six items (e.g., “You have a certain body weight, and you can’t really do much to change it”; *α* = 0.920) on a scale from 1 (*strongly agree*) to 6 (*strongly disagree*). Items were reverse scored as necessary so that higher scores indicate stronger growth mindsets. *Intelligence mindsets* (Dweck, 2000) were assessed as the average of six items (e.g., “Your intelligence is something about you that you can’t change very much”; α = 0.942) on a scale from 1 (*strongly agree*) to 6 (*strongly disagree*). Items were reverse scored as necessary so that higher scores indicate stronger growth mindsets.

#### Behavioral obligation manipulation check

Immediately following exposure to the behavioral obligation message, participants were asked the following question, “According to this information, if you learn that you are at high risk of prediabetes, what should you do?” Response options included, “Make an appointment with a physician,” “I have not been given this information yet,” and “Don’t know/not sure.” Participants in the high behavioral obligation condition were coded as responding correctly if they selected, “Make an appointment with a physician” and as responding incorrectly if they selected a different response. Participants in the low behavioral obligation condition were coded as responding correctly if they selected, “I have not been given this information yet” and as responding incorrectly if they selected a different response.

#### Demographic characteristics

Participants also reported demographic characteristics, including age, gender, race and ethnicity, and education (see Table 1). For analyses, gender was coded as male = 1 and female = 0; and race was coded as White = 1 and members of a different racial group = 0. Education was recoded into four categories: completed high school or less = 1, completed vocational/technical training or some college = 2, college graduate = 3, and postgraduate degree = 4.

### Overview of analyses

We first examined participant characteristics, the proportion of correct responses to the behavioral obligation manipulation check, and correlations among variables. This included examining bivariate associations among health mindsets and avoidance intentions and behavior. We then tested the interactive effects of physical health mindsets and high and low behavioral obligation on avoidance intentions and behavior. This was tested using hierarchical regression analyses with covariates of age, gender, race, and education entered in Step 1, the mean-centered main effect of health mindsets and the main effect of behavioral obligation in Step 2, and the interaction of mindsets and behavioral obligation in Step 3. Regression analyses were linear for the continuous outcome of avoidance intentions and logistic for the dichotomous outcome of avoidance behavior. Age, gender, education, and race were chosen as covariates because they are commonly controlled for in psychological studies of this type. Similar to Study 1, because of a high rate of incorrect responses to the behavioral obligation manipulation check, analyses involving behavioral obligation were conducted based on (1) randomly assigned condition, and (2) how participants responded to the manipulation check. Next, we examined the bivariate correlations among the five health agency belief constructs and with avoidance intentions and behaviors. We also examined the correlations among physical health, weight, and intelligence mindsets. Then, we tested each of the five agency beliefs as a predictor of avoidance intentions and behavior in separate regression analyses controlling for age, gender, race, and education. For any agency belief that was significantly associated with intentions or behavior, this analysis was also run controlling for health mindsets.^5^

### Study 2 results

#### Preliminary analyses

Table 1 provides demographic characteristics of the sample. On average, participants’ risk for prediabetes was low, *M* = 2.40, *SD* = 1.61, and participants endorsed growth mindsets, *M* = 4.42 out of 6, *SD* = 1.03. Participants’ intentions to avoid their prediabetes risk was about at the midpoint of the scale, *M* = 2.79 out of 5, *SD* = 1.39, and 43.13% (*n* = 317) of participants avoided learning their risk of prediabetes. As shown in Table 2, health mindsets were not significantly associated with avoidance intentions, *r*(735) = − 0.06, *p* = .062, but stronger growth mindsets were associated with lower avoidance behavior, *r*(735) = − 0.07, *p* = .037.

**Table 2:** Correlations among health agency beliefs, mindsets, and avoidance outcomes, Study 2

	1	2	3	4	5	6	7	8	9
1. Health mindsets	−								
2. Weight mindsets	0.61**	−							
3. Intelligence mindsets	0.29**	0.27**	−						
4. Internal health locus of control	0.30**	0.17**	0.15**	−					
5. Chance health locus of control	− 0.39**	− 0.34**	− 0.18**	− 0.22**	−				
6. Health self-efficacy	0.28**	0.24**	0.14**	0.38**	− 0.21**	−			
7. Health fatalism	− 0.43**	− 0.36**	− 0.17**	− 0.26**	0.76**	− 0.31**	−		
8. Genetic determinism	− 0.25**	− 0.23**	− 0.11**	− 0.06	0.51**	− 0.06	0.41**	−	
9. Avoidance intentions	− 0.06	− 0.01	− 0.08*	− 0.14**	− 0.06	− 0.10**	− 0.05	− 0.08*	−
10. Avoidance behavior ^a^	− 0.07^†^	− 0.06	− 0.03	− 0.09*	0.02	− 0.01	0.04	− 0.03	0.61**
Mean	4.42	4.80	3.90	4.29	3.04	3.07	2.42	3.32	2.79
Standard deviation	1.03	1.11	1.30	0.73	0.95	0.62	0.78	0.63	1.39
Possible range	1–6	1–6	1–6	1–6	1–6	0–4	1–5	1–5	1–5

Note. *n* = 735 for all analyses. Following our preregistered plan, all analyses reported are two-tailed other than the associations between health mindsets and avoidance intentions and avoidance behavior, which are one-tailed

^a^ Avoided risk = 1, learned risk = 0

*p < .05, two-tailed. **p < .01, two-tailed. ^†^*p* < .05, one-tailed

In the high behavioral obligation condition, 95.36% (349 of 366) of participants correctly responded that they would need to make an appointment with a physician. In the low behavioral obligation condition, 72.90% (269 of 369) correctly responded that they had not been given information yet about what to do if they were at high risk of prediabetes. Consistent with Study 1, given the larger number of participants answering incorrectly than anticipated (*n* = 117), we retained all participants in analyses.

#### Associations of health mindsets and behavioral obligation with avoidance

A linear regression testing the effect of health mindsets, behavioral obligation condition, and their interaction on information avoidance intentions was conducted controlling for age, gender, education, and race. Health mindsets were not significantly associated with intentions to avoid learning one’s risk of prediabetes, *B *= − 0.12, *SE* = 0.07, *p* = .081, 95% CI [− 0.26, 0.02]. Likewise, behavioral obligation condition did not predict avoidance intentions, main effect: *B* = 0.07, *SE* = 0.10, *p* = .474, 95% CI [− 0.13, 0.27], nor was there an interaction of health mindsets and behavioral obligation, *B* = 0.07, *SE* = 0.10, *p* = .513, 95% CI [− 0.13, 0.26]. When analyses were rerun based on how participants responded to the behavioral obligation manipulation check, rather than randomly assigned condition, all effects remained nonsignificant.

A logistic regression testing the effect of health mindsets, behavioral obligation condition, and their interaction on information avoidance behavior was conducted controlling for age, gender, education, and race. Health mindsets were not significantly associated with avoidance of prediabetes risk information, *OR* = 0.88, *p* = .226, 95% CI [0.72, 1.08]. Participants in the high behavioral obligation condition avoided at a greater rate than those in the low behavioral obligation condition, *OR* = 1.42, *p* = .022, 95% CI [1.05, 1.90]. Health mindsets and behavioral obligation did not interact to predict avoidance behavior, *OR* = 0.96, *p* = .759, 95% CI [0.72, 1.28]. When analyses were rerun based on how participants responded to the behavioral obligation manipulation check, rather than randomly assigned condition, there was no longer a significant effect of behavioral obligation condition on avoidance behavior, *OR* = 1.34, *p* = .066, 95% CI [0.98, 1.83]. The main effect of mindsets and the interaction remained nonsignificant.

#### Associations among health agency beliefs and mindsets

As shown in Table 2, in support of our hypothesis, health mindsets were significantly associated with all health agency belief constructs. Correlations among health mindsets and agency beliefs ranged from *r*(735) = − 0.25 for genetic determinism to *r*(735) = − 0.43 for fatalism, suggesting health mindsets are related to but distinct from these constructs. Health mindsets were also significantly associated with weight mindsets, *r*(735) = 0.61, and contrary to our hypothesis, significantly associated with intelligence mindsets, *r*(735) = 0.29, all *p*s < 0.001.

#### Associations among health agency beliefs and avoidance

As shown in Table 2, greater internal health locus of control beliefs, *r*(735) = − 0.14, *p* < .001, greater health self-efficacy, *r*(735) = − 0.10, *p* = .007, and greater genetic determinism beliefs, *r*(735) = − 0.08, *p* = .026, were associated with lower avoidance intentions.

As shown in Table 3, we ran a series of hierarchical linear regressions to test whether agency beliefs were associated with avoidance intentions after controlling for age, gender, race, and education. Each agency belief was entered as a predictor in a separate regression analysis. Consistent with bivariate analyses, participants with lower internal health locus of control beliefs, lower health self-efficacy, and lower genetic determinism beliefs reported greater avoidance intentions. Also consistent with bivariate analyses, health mindsets, chance health locus of control, and fatalism remained unassociated with avoidance intentions after controlling for demographic factors. Following the preregistered plan, we ran additional hierarchical regressions to test whether any significant predictors of avoidance (i.e., internal health locus of control, health self-efficacy, and genetic determinism) remained significant after controlling for health mindsets. All three variables remained significant predictors of avoidance intentions when controlling for health mindsets on an earlier step in the regression: internal health locus of control: *B *= − 0.25, *SE* = 0.07, *p* < .001, 95% CI [− 0.39, − 0.10]; health self-efficacy: *B *= − 0.18, *SE* = 0.09, *p* = .043, 95% CI [− 0.35, − 0.01]; genetic determinism: *B *= − 0.21, *SE* = 0.08, *p* = .011, 95% CI [− 0.38, − 0.05]. Of note, in these models, health mindsets were only a significant predictor when genetic determinism was in the model (*B *= − 0.12, *SE* = 0.05, *p* = .018, 95% CI [− 0.22, − 0.02]), but not when internal health locus of control or health self-efficacy were included as predictors.

**Table 3:** Regression results for information avoidance intentions and behavior, Study 2

Variable	Avoidance Intentions								Avoidance Behavior			
	B	95% CI for B		SE B	*p*	β	R^2^		OR	95% CI for B		*p*
		LL	UL							LL	UL	
Step 1												
Age	− 0.01	− 0.02	0.004	0.01	0.246	− 0.04			0.99	0.97	1.00	0.062
Race	0.33	0.08	0.58	0.13	0.009	0.10			1.30	0.90	1.88	0.167
Education	− 0.12	− 0.24	< 0.001	0.06	0.050	− 0.07			0.88	0.73	1.04	0.138
Gender	0.19	− 0.01	0.40	0.11	0.065	0.07			1.19	0.88	1.61	0.267
Step 2: Individual entry												
Health mindsets	− 0.09	− 0.19	0.01	0.05	0.072	−0.07	0.03		0.87	0.75	0.99	0.049
Internal health locus of control	− 0.26	− 0.40	− 0.13	0.07	<0 .001	− 0.14	0.04		0.77	0.62	0.94	0.010
Chance health locus of control	− 0.08	− 0.19	0.02	0.05	0.129	− 0.06	0.03		1.04	0.89	1.22	0.612
Health self-efficacy	− 0.21	− 0.37	− 0.04	0.08	0.014	− 0.09	0.03		1.02	0.80	1.30	0.864
Health fatalism	− 0.07	− 0.20	0.06	0.07	0.322	− 0.04	0.03		1.12	0.93	1.36	0.242
Genetic determinism	− 0.16	− 0.32	− 0.01	0.08	0.044	− 0.07	0.03		0.91	0.72	1.15	0.430

Note. All regression analyses controlled for age, race, education, and gender in Step 1. The “Step 2: Individual entry” analyses show the results of six separate regression analyses in which each predictor was entered as the only predictor in Step 2

Regarding avoidance behavior, bivariate analyses indicated that participants with greater (vs. lower) internal health locus of control beliefs, *r*(751) = − 0.091, *p* = .014 were less likely to avoid learning their risk. No other health agency beliefs were associated with avoidance behavior.

As shown in Table 3, a series of hierarchical logistic regressions tested whether agency beliefs were associated with avoidance behavior after controlling for age, gender, race, and education. Each agency belief was entered as a predictor in a separate regression analysis. Results were consistent with bivariate analyses: participants with stronger growth mindsets and internal health locus of control beliefs were less likely to avoid. When also controlling for health mindsets on an earlier step in the logistic regression analysis, internal health locus of control remained a significant predictor of avoidance behavior, *OR* = 0.80, *p* = .039, 95% CI [0.64, 0.99]. Health mindsets were no longer a significant predictor when internal health locus of control was in the model, *OR* = 0.91, *p* = .213, 95% CI [0.78, 1.06].

### Study 2 discussion

In Study 2, we examined associations among agency beliefs—including health mindsets—and information avoidance. Whereas health mindsets were not significantly associated with avoidance intentions, there was some evidence that participants with a weaker health growth mindset were more likely to avoid learning their prediabetes risk (although the strength of this relationship depended on which other factors were included in the statistical model). Of note, only 9.25% of participants held a “true” fixed mindset (i.e., scored between 1 and 3 on the 6-point scale), which may have lessened the likelihood of finding significant associations among health mindsets and avoidance. The strongest and most robust predictor of both avoidance intentions and behavior across analyses was internal health locus of control. The finding that individuals who believe they have more control over their health are less likely to engage in information avoidance is consistent with prior literature (Orom et al., 2021).

## Discussion

When faced with negative feedback, people with weaker growth mindsets—that is, who believe attributes are unchangeable and unimprovable—are more likely to respond defensively (Nussbaum & Dweck, 2008). Learning whether one is at risk of disease may be a similarly threatening experience that carries the possibility of responding defensively by avoiding the information. In the case of prediabetes risk, individuals who learn that they are at increased risk of developing prediabetes can use this information to make behavioral changes. However, if a person believes they cannot do much to change their physical health, they may be motivated to avoid the information. Across two studies, we tested the hypothesis that individuals with more fixed mindsets about their physical health would report greater intentions to avoid their prediabetes risk and would be more likely to avoid learning their risk when given an opportunity to do so. Contrary to this hypothesis, the pattern of results across both an experiment (Study 1) and a cross-sectional correlational study (Study 2) largely indicated that physical health mindsets were *not* associated with information avoidance intentions or behavior.

### Null effects of health mindsets on information avoidance

In Study 1, there was no effect of manipulated health mindsets on information avoidance intentions or behavior despite the successful manipulation of mindsets. In Study 2, measured (not manipulated) health mindsets were not associated with avoidance intentions, and the association of mindsets with behavior varied between significant and nonsignificant depending on which other variables were included in the analytic model. Taken together, there was little support for an association of health mindsets with health information avoidance across studies and analyses. One possible explanation for the null findings is that most people already believe their health is something they can change with effort. In both studies, most participants endorsed growth mindsets, perhaps making it difficult to detect differential effects of growth vs. fixed mindsets on information avoidance. It is also possible that health mindsets may not be a key psychological construct in the domain of physical health, at least with respect to learning information about one’s personal risk. Perhaps mindsets matter more in specific health contexts than for physical health generally. For example, parents holding more growth beliefs about weight were more likely to choose to receive information about environmental factors that contribute to their children’s weight and eating habits (Hagerman et al., 2022). In another study, people with greater growth weight mindsets were less likely to avoid learning their body composition (i.e. body fat, weight, and muscle composition; Hagerman et al., 2023). Although these findings contrast with those of the current studies, the differences may be attributable to the assessment of weight specifically versus health more generally.

The level of threat evoked by prediabetes within the populations from which we sampled may also explain the largely null association of health mindsets with information avoidance. Mindset theory suggests that individuals with a fixed mindset tend to respond in ways that allow them to *prove* themselves vs. *improve* themselves (Dweck et al., 1995b). Someone with a fixed mindset about health who is certain they are not at risk for prediabetes would have no reason to avoid that information. Similarly, threat may be an important precursor for an individual to respond defensively. The level of threat may also explain why health mindsets were only associated with avoidance behavior in the general adult sample (in some analyses); this sample was older and had, on average, slightly higher risk of prediabetes.

### Additional health agency constructs

A secondary aim, which we addressed in Study 2, was to examine the relationships among health mindsets and other constructs, including both weight and intelligence mindsets. Earlier research established mindsets as being contextually distinct (Dweck et al., 1995a). That is, a person can believe their intelligence is something that can change, but their health less so. Here, we found that participants who endorsed the belief that their health is malleable typically endorsed similar beliefs in both intelligence and weight domains, with health and weight mindsets more strongly correlated than health and intelligence mindsets.

In addition to examining other mindset domains, in Study 2 we examined relationships among health mindsets and other health agency beliefs. For example, genetic determinism assesses beliefs about the extent to which genes affect one’s physical health (Parrott et al., 2012), and it is logical to presume that if someone believes their health is not malleable, they may attribute more of their health to a factor such as their genetic makeup. Our correlational findings provided initial evidence that health mindsets are distinct from other health agency constructs, including health locus of control, health self-efficacy, fatalism, and genetic determinism.

One finding that deserves future consideration is the correlations amongst the other health agency measures. Specifically, chance health locus of control (CHLC) was strongly correlated with fatalism (*r* = .76), and moderately strongly correlated with genetic determinism (*r* = .51). The strong correlation between CHLC and fatalism is not surprising: both measures assess cognitions regarding the role of luck and fate in determining one’s health. For example, one item in the CHLC scale is, “No matter what I do, if I am going to get sick, I will get sick,” whereas one item in the fatalism scale (predetermination subscale) is, “If someone is meant to get a serious disease, they will get it no matter what they do.” A strong correlation between two measures assessing a similar, if not the same, construct risks contributing to what is referred to as the jangle fallacy. The jangle fallacy occurs when two measures are labeled differently, suggesting that they assess different constructs, when in fact they assess the same construct (Gonzalez et al., 2021). Future work may consider using methods such as structural equation modeling to identify latent constructs concerning agency beliefs.

Contrary to the largely null effects for health mindsets, greater internal health locus of control—that is, beliefs that one has more personal control over their health—was consistently associated with lower intentions to avoid learning health information and lower rates of avoidance behavior. Similarly, in another study internal health locus of control was significantly associated with a self-reported preference to avoid diabetes risk information (Orom et al., 2021). Thus, internal health locus of control is deserving of future research attention with respect to health information avoidance.

### Behavioral obligation

Prior research suggests people are more likely to avoid when learning the information may require them to take additional action (Howell & Shepperd, 2013; Sweeny et al., 2010). Inconsistent with this, in the present studies we generally found that participants who were told they would need to follow up with a provider for additional testing if they were at high risk were *not* more likely to avoid than participants who were not given such information. However, given the number of participants in both studies who incorrectly answered the manipulation check question, caution should be taken when interpreting the results of the behavioral obligation manipulation. It is not entirely clear why participants answered incorrectly at the rate that they did because in Study 1, more participants in the high behavioral obligation condition answered incorrectly, whereas in Study 2, more participants in the low behavioral obligation condition answered incorrectly. One possible explanation for Study 2 is that participants may have held pre-existing beliefs about the implications of being at risk for prediabetes. That is, they may have assumed that additional action, such as following up with a medical provider, would be necessary if an online risk calculator indicated high risk. Nevertheless, most participants (82.7% in Study 1; 84.1% in Study 2) did respond correctly to the behavioral obligation manipulation check question, suggesting that the null effect of this manipulation on avoidance cannot be completely explained by incorrect responses to the manipulation check question. Perhaps most people believed they would be at lower risk (which was indeed the case for both samples) and thus, did not expect they would need to follow up with a medical provider.

### Implications and future research directions

People are faced with the prospect of learning health information in multiple settings. In the present study, people could learn their disease risk through an online calculator. However, people also have opportunities to learn or avoid health information as they go about their daily lives (Foust & Taber, 2024). For example, people can monitor and track their health using their mobile device. In one study, people with greater internal health locus of control beliefs were more willing to use these tools to monitor their health (Bennett et al., 2017), suggesting the relationship between internal health locus of control beliefs and willingness to engage with health information exists beyond the specific context of personalized health risk information. In general, public health interventions might seek to increase people’s beliefs that their own actions play a role in their health outcomes. Interventions may also seek to increase people’s knowledge about and skills regarding improving their health outcomes, which may influence health locus of control beliefs.

The opportunity to learn or avoid health information is also relevant to clinical populations. Although more research is needed on how to effectively increase internal health locus of control beliefs, medical providers’ discussions with their patients may represent a low-cost opportunity to target such beliefs. For example, a medical provider who recommends that a patient with diabetes should monitor their blood sugar levels at home may discuss specific dietary options or how to overcome potential barriers to increase perceived control.

That said, control beliefs are also likely informed by a person’s broader social environment, including systemic barriers such as medical care access, reliable transportation, and time. Amongst a sample of Latino adults, a lack of health care utilization was better explained by a lack of medical care access than locus of control beliefs (De Jesus & Xiao, 2014). More research is needed on whether systemic barriers such as these better explain the decision to avoid health information than internal health locus of control beliefs, as well as how these factors interact. In some instances, it may be more appropriate to focus efforts on minimizing systemic barriers rather than attempting to change locus of control beliefs.

### Strengths and limitations

One strength of the current work is the converging evidence across two studies using different designs and samples. While it is possible the null findings in Study 1 are attributable to young adults not perceiving prediabetes as relevant due to their age, our finding that mindsets were not associated with avoidance of prediabetes risk information was largely replicated in Study 2, suggesting age alone does not explain the null findings. Another strength is that we measured avoidance behavior: What people think they would do when given the opportunity to learn their risk versus what they actually do may differ, and we assessed participants’ decisions to avoid when given a real opportunity to receive personalized risk information.

The studies have some limitations. First, participants may have correctly concluded that their risk of prediabetes was low and therefore believed that learning their personalized disease risk estimate would not be necessary. However, the prediabetes infographic noted that most of the 1 in 3 U.S. adults who have prediabetes are unaware of their status, which could have motivated participants to learn their risk even if they expected it to be low. Alternatively, if participants avoided their risk not because they felt threatened, but due to logistic reasons such as a lack of time or money (see O’Brien et al., 2024), mindsets may not have influenced information avoidance in these studies.

In addition, about 80% of Study 1 participants were female, which could have impacted study results given some evidence of gender differences in online information seeking behaviors (e.g., Rowley et al., 2017). However, given that other research has not found significant gender differences in the tendency to avoid health information (Howell & Shepperd, 2016), we believe it is unlikely that the gender make-up of Study 1 heavily impacted the overall conclusions we drew about the current findings.

The way in which we assessed information avoidance may limit generalizability of the current findings. In both studies, participants were required to follow a link to an external website to learn their risk. Participants may have disliked the idea of following a link to an external website or distrusted the CDC; findings may have differed if participants completed the risk calculator within the survey itself or if the source of the risk calculator was not provided (see O’Brien et al. 2024, concerning self-reported reasons for avoiding health risk information in the datasets reported here as well as in other datasets).

There are also other factors that were not assessed as part of the current study that could have influenced the results. For example, it would be advantageous for future research to examine the role of prior experiences with health information and with the medical care system as potentially interacting with locus of control beliefs to influence information avoidance.

## Conclusion

Physical health mindsets are a relatively new application of mindset theory. Thus, it is important to understand when, and for whom, health mindsets may predict health beliefs, decisions, and behavior. The present findings can contribute to future meta-analyses on the topic. There are also implications for the information avoidance literature. Researchers have aimed to understand individual differences that predict health information avoidance. Our findings suggest that health mindsets may not be as consequential in the context of information avoidance as other individual differences such as internal health locus of control beliefs. Future research may focus on people’s beliefs about how their own actions can impact their health.

## Electronic supplementary material

Below is the link to the electronic supplementary material.

Supplementary Material 1

## Data Availability

Data and syntax are available upon request from the author.

## Footnotes

1 Although we pre-registered an a prior power analysis that supported a sample size of 218 respondents, we were unable to recreate these numbers at the time of manuscript writing.

2 We preregistered excluding participants who incorrectly answered the behavioral obligation manipulation. Upon further reflection, we opted to retain all participants in analysis given this item was included as a manipulation rather than comprehension check.

3 We preregistered the following a prior power analysis: “An a priori power analysis in G*Power (Faul, 2009) indicated that a sample of 770 participants would allow us to detect a small effect size of 0.02 at *p* < .05 with .085 power using a linear regression with up to 15 predictors.” This power analysis was used to guide the sample size during data collection, but we did not preregister any regressions with 15 predictors.

4 The Multidimensional Health Locus of Control scales (Wallston et al., 1978) also includes the Powerful Others subscale that assesses the extent to which people believe others such as health professionals control their health. Because the internal and chance health locus of control subscales are most relevant to growth and fixed mindsets, the Powerful Others subscale was not included in analyses.

5 We preregistered that we would simultaneously test the five agency beliefs as predictors of avoidance. However, we do not report results of any analyses with these variables as simultaneous predictors of either avoidance intentions or behavior due to significant multicollinearity concerns (stemming from the high intercorrelations among the health agency beliefs).

## Materials availability

The study design, hypotheses, and analytic plan were pre-registered on Open Science Framework (OSF; Study 1: bit.ly/Study1mindsets, Study 2: bit.ly/Study2Mindsetsoriginal and bit.ly/Study2Mindsetsupdated).
