# Supplementary material for: Physical health mindsets and information avoidance
Source: J Behav Med. 2024 Sep 21;47(6):1052–66. doi: 10.1007/s10865-024-00514-1 (PMC11499518; doi:10.1007/s10865-024-00514-1)
Supplement: Supplementary file 1 — Supplementary Material 1 [file 10865_2024_514_MOESM1_ESM.docx]

**Electronic Supplemental Materials**

**Physical Health Mindsets and Information Avoidance**

*Journal of Behavioral Medicine*

**Index:**

*Materials are presented in the order they appear in the main manuscript*

1. Pre-registered analyses not central to the present study
2. Sample exclusions, Study 1
3. Prediabetes Infographic, Study 1 and Study 2
4. Objective risk items
5. Health Mindset Manipulation, Study 1
   1. Growth Mindset Condition
   2. Fixed Mindset Condition
6. Behavioral Obligation Manipulation, Study 1
7. Sample exclusions, Study 2
8. Behavioral Obligation Manipulation, Study 2
9. Pre-registered analyses not central to the present study

**Hypotheses**

We hypothesized that among the individuals who did not avoid learning information about their health, those with stronger (versus weaker) growth health mindsets would spend more time engaging with the risk information. Because prior research has found evidence to suggest self-efficacy mediates the relationship between mindsets and health outcomes (Ehrlinger et al., 2017; Orvidas et al., 2018), we also hypothesized that health self-efficacy would mediate the relationship between health mindsets and avoidance.

**Method**

***Time Spent Engaging with Risk Information***

Embedded data in Qualtrics allowed us to assess how much time elapsed between participants deciding to learn their risk and continuing with the survey. These data were used as a proxy for how much time participants spent attending to the risk information about diabetes.

**Study 1 Results**

We used a *t*-test to test the hypothesis that participants in the growth mindset condition spent more time engaging with the risk information than those in the fixed mindset condition. Among participants who learned their risk, those in the growth mindset condition did not spend more time engaging with the risk information, *M*=81.54 seconds, *SD*=24.59, than those in the fixed mindset condition, *M*=80.25, *SD*=25.34; *t*(142)=.310, *p*=.757. Thus, this hypothesis was not supported.

**Study 2 Results**

***Health Mindsets and Time Spent Engaging with Risk Information***

We used correlations to examine whether health mindsets were associated with time spent engaging with the risk information. On average, participants spent about 1 minute with the risk information (*M* = 61.37 seconds, *SD* = 48.17, in seconds). A partial correlation analysis tested the hypothesis that among participants who did learn their risk, those with stronger growth mindsets would spend more time engaging with the risk information, controlling for age, race, gender, and education. Our hypothesis was not supported, *r*(412)=-.02, *p*=.362, nor was the relationship significant when conducting a zero-order correlation between time spent and health mindsets, *r*(418)=-.02, *p*=.650.

***Mediation Analyses***

We conducted mediation analyses using Hayes’ PROCESS macro to test our hypothesis that self-efficacy would mediate the relationship between health mindsets and avoidance intentions and behavior. Health mindsets were significantly associated with self-efficacy, *B*=0.17, *SE*=0.02, 95% CI [0.12, 0.21], *β*=0.28, *p*<.001 and self-efficacy was significantly associated with avoidance intentions, *B*= -0.20, *SE*=0.09, 95% CI [-0.37, -0.03], *β*= -0.09, *p*=.0184. The direct effect of health mindsets on avoidance intentions was not significant, *B*= -0.04, *SE*=0.05, 95% CI [-0.14, 0.06], *β*= -0.03, *p*=.403 but the indirect effect was, *B*= -0.03, *SE*=0.02, 95% CI [-0.07, -0.01], *β*=0.02. For avoidance behavior, self-efficacy was not significantly associated with avoidance behavior, *B*=0.03, *SE*=0.13, 95% CI [-0.22, 0.27], *p*=.819. Likewise, the direct effect of health mindsets on avoidance behavior was not significant, *B*= -0.13, *SE*=0.08, 95% CI [-0.28, 0.01], *p*=.075 nor was the indirect effect significant, *B*=0.005, *SE*=0.02, 95% CI [-0.04, 0.05]. The results for avoidance behavior were inconsistent with our hypothesis. In support of our hypothesis, self-efficacy mediated the relationship between health mindsets and avoidance intentions, although the effect sizes were small. However, in contrast to the hypothesis, there was no evidence that self-efficacy mediated an association between health mindsets and avoidance behavior.

**Additional Analyses**

The following pre-registered analyses were determined to be beyond the scope of the present studies and were not conducted for the present studies. We ultimately conducted data analysis for the two studies simultaneously rather than sequentially. Of note, in the pre-registration, the correlational MTurk study is referred to as Study 1 (referred to as Study 2 in the present manuscript) and the experimental student study is referred to as Study 2 (referred to as Study 1 in the present manuscript). Text from pre-registration:

- We plan to conduct data analysis for the first study prior to analyzing data from this study, and will use Study 1 to inform secondary analyses for Study 2. For example, in Study 1 we will examine whether specific prediabetes risk factors (e.g., family history, blood pressure, age, race, physical activity levels, gender, and BMI) are associated with information avoidance. Risk factors associated with information avoidance in Study 1 may be tested as moderators in Study 2.
- We also plan to conduct analyses specifically among the subset of respondents who opt to learn their risk concerning rejection or disagreement with risk estimates.

1. Sample exclusions, Study 1

Participants were excluded if they incorrectly answered the prediabetes infographic questions (*n*=27), if they incorrectly answered the mindset reading comprehension check question (*n*=9), if they misunderstood the risk calculator instructions, such as missing the link to the risk calculator website (*n*=11), if they had missing data for the health mindset measure (*n*=1), or if they had missing data for the avoidance measures (*n*=1). Participants were also excluded if the risk calculator website was not working/unavailable while they were taking the study (*n*=4) or if they reported suspicion of the purpose of the study during the debriefing process (*n*=9). Finally, one participant was excluded due to learning about the study prior to participating. Exclusion criteria were not mutually exclusive.

1. Prediabetes infographic shown to participants, Study 1 and Study 2


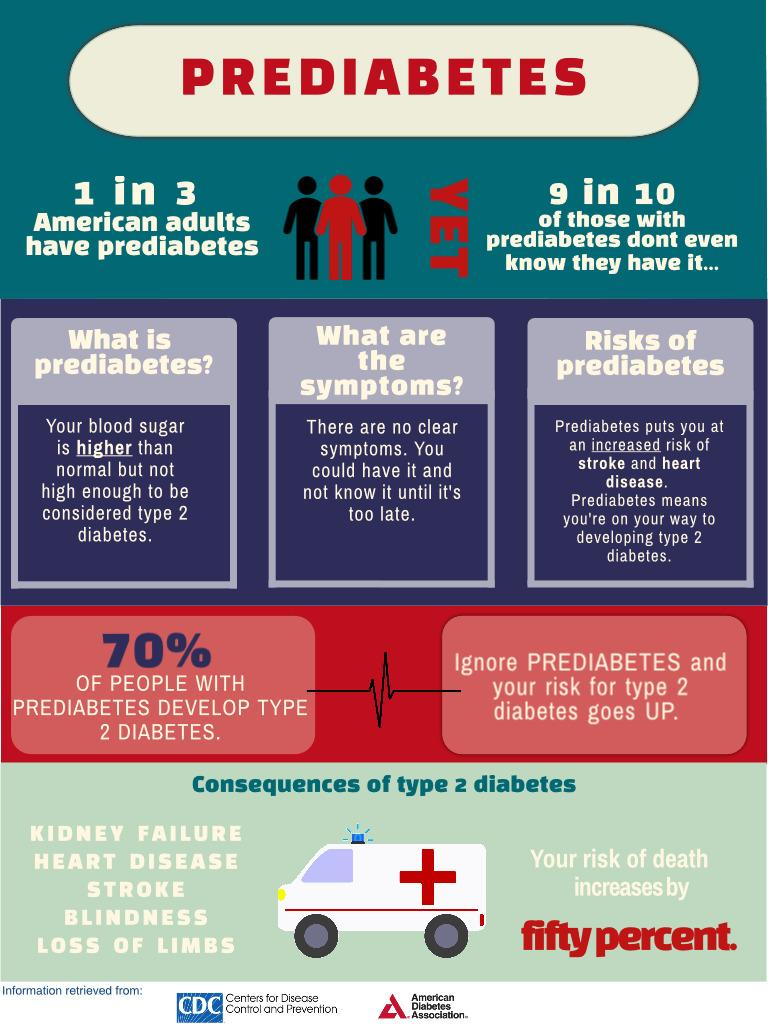


1. Prediabetes risk calculator items, Study 1 and Study 2

https://www.cdc.gov/prediabetes/risktest/index.html

| **Height** | **Weights (lbs)** | | |
| --- | --- | --- | --- |
| 4’10” | 119-142 | 143-190 | 191+ |
| 4’11” | 124-147 | 148-197 | 198+ |
| 5’0” | 128-152 | 153-203 | 204+ |
| 5’1” | 132-157 | 158-210 | 211+ |
| 5’2” | 136-163 | 164-217 | 218+ |
| 5’3” | 141-168 | 169-224 | 225+ |
| 5’4” | 145-173 | 174-231 | 232+ |
| 5’5” | 150-179 | 180-239 | 240+ |
| 5’6” | 155-185 | 186-246 | 247+ |
| 5’7” | 159-190 | 191-254 | 255+ |
| 5’8” | 164-196 | 197-261 | 262+ |
| 5’9” | 169-202 | 203-269 | 270+ |
| 5’10” | 174-208 | 209-277 | 278+ |
| 5’11” | 179-214 | 215-285 | 286+ |
| 6’0” | 184-220 | 221-293 | 294+ |
| 6’1” | 189-226 | 227-301 | 302+ |
| 6’2” | 194-232 | 233-310 | 311+ |
| 6’3” | 200-239 | 240-318 | 319+ |
| 6’4” | 205-245 | 246-327 | 328+ |
|  | **1 Point** | **2 Points** | **3 Points** |

1. How old are you?
   1. Younger than 40 years (0 points)
   2. 40 - 49 years (1 point)
2. 50 - 59 years (2 points)
   1. 60 years or older (3 points)
3. Are you a man or a woman?
   1. Man (1 point)
   2. Woman (0 points)
4. If you are a woman, have you ever been diagnosed with gestational diabetes?
   1. Yes (1 point)
   2. No (0 points)
5. Do you have a mother, father, sister, or brother with diabetes?
   1. Yes (1 point)
   2. No (0 points)
6. Have you ever been diagnosed with high blood pressure?
   1. Yes (1 point)
   2. No (0 points)
7. Are you physically active?
   1. Yes (0 points)
   2. No (1 point)
8. What is your weight category?
9. Health Mindset Manipulation, Study 1

*Growth Mindset Condition*

**Harvard Professor’s Latest Research Findings About Physical Health**

By: David Schumann

| We are all familiar with the age-old debate: should we eat that extra piece of cake, or should we choose the salad instead?    Researchers, not just regular people, have been asking themselves the same questions – and have been trying to find answers. Dr. Gregory Pierce has been studying people’s health behaviors for the entirety of his career at Harvard University. New research findings from Pierce and his colleagues suggests that overall health status in adulthood tends to be extremely flexible, and that engaging in health promoting behaviors can make a lot of difference in one’s health.    In several different studies conducted in various locations around the United States, Pierce followed individuals who were aged 18 to 70 and studied their health-related behaviors, such as exercise frequency, vegetable and fruit consumption, hours of sleep, and stress levels. He also measured how many times these adults got sick | every year, their blood pressure, cholesterol, body fat percentage, and how many of these adults were diagnosed with a major illness such as cancer. Pierce found that, in general, engaging in health promoting behaviors made a substantial difference, for the better, in the physical health status of these adults.    Pierce explained that once we pass adolescence, “our genetic makeup, our DNA, does not really affect our health status.” He found that adults who ate healthy diets and exercised regularly were in much better health than those who partied regularly, ate fast food often, and exercised infrequently – regardless of genetic makeup. Perhaps more promising, those who engaged in healthy behaviors were less likely to be diagnosed with both cancer and diabetes. In essence, health status in adulthood is a direct reflection of one’s lifestyle – if you change your lifestyle, you can really change your health a lot. The choice is entirely yours. | Pierce continued, “It is clear that genetic predispositions can be overridden by day to day routines, such as exercising, getting enough sleep, and trying to eat better. As a result, your health can change dramatically from one year to the next, in tandem with your lifestyle.”    In light of these findings, perhaps we do have an answer to that famous question: choose the salad as often as you can. Making healthier decisions will make all the difference in your health. A little effort goes a long way, and research shows that it’s worth it in the long run to give up that extra piece of cake. |
| --- | --- | --- |

B. *Fixed Mindset Condition*

**Harvard Professor’s Latest Research Findings About Physical Health**

By: David Schumann

| We are all familiar with the age-old debate: should we eat that extra piece of cake, or should we choose the salad instead?    Researchers, not just regular people, have been asking themselves the same questions – and have been trying to find answers. Dr. Gregory Pierce has been studying people’s health behaviors for the entirety of his career at Harvard University. New research findings from Pierce and his colleagues suggest that overall health status in adulthood tends to be extremely stable, which is a result of genetic predisposition.    In several different studies conducted in various locations around the United States, Pierce followed individuals who were aged 18 to 70 and studied their health-related behaviors, such as exercise frequency, vegetable and fruit consumption, hours of sleep, and stress levels. He also measured how many times these adults got sick every year, their blood pressure, | cholesterol, body fat percentage, and how many of these adults were diagnosed with a major illness such as cancer. Pierce found that, in general, health status of these adults did not change as a function of engagement in ‘health promoting’ behaviors.    Pierce explained that once we pass adolescence, “our genetic makeup, our DNA, plays a much larger role in determining our health than we thought.” He found that adults who were genetically healthier could party regularly, eat fast food often, exercise infrequently, and still be in excellent health. On the other hand, those who had less resilient genes could eat very healthy diets and exercise regularly but would still frequently encounter health complications. Perhaps most surprisingly, those who engaged in healthy behaviors and those who did not were equally as likely to be diagnosed with both cancer and diabetes. In essence, health status during adulthood seems to be fixed. | Pierce continued, “I’m not saying that if you exercise you won’t get more fit, because you will. What I’m saying is that your physical health status does not depend on your ‘health promoting’ behaviors during adulthood – it depends on your genetics, and genetics are a part of you that you cannot change.”    In light of these findings, perhaps we do have an answer to that famous question: always choose the cake. It was once believed making “healthier” decisions would improve our health. New research is now suggesting that this isn’t true. So, what’s the point? You might as well enjoy yourself. |
| --- | --- | --- |

1. Behavioral Obligation Manipulation, Study 1

| High behavioral obligation condition | Low behavioral obligation condition |
| --- | --- |
| Due to the increasing number of younger adults getting diagnosed with prediabetes (and subsequently type 2 diabetes), the Department of Psychological Sciences at Kent State University has teamed up with the DeWeese Health Center to promote awareness of the disease.  The American Diabetes Association has developed an accurate way to calculate your risk of prediabetes. You can learn your risk by answering several questions about yourself. If you score higher than a certain number, you are likely to have prediabetes and are at high risk of type 2 diabetes. **If you choose to calculate your risk today, and the calculator indicates you are high risk of prediabetes, you will be contacted by a physician from DeWeese Health Center to schedule blood work for a definite diagnosis.** | The American Diabetes Association has developed an accurate way to calculate your risk of prediabetes. You can learn your risk by answering several questions about yourself. If you score higher than a certain number, you are likely to have prediabetes and are at high risk of type 2 diabetes. **If you choose to calculate your risk today, and the calculator indicates you are high risk of prediabetes, you will be given information about what to do next.** |

1. Sample exclusions, Study 2

We preregistered that we would collect data from 800 participants to account for exclusions. However, after collecting data from 803 participants, 177 respondents were excluded due to poor quality data, based on reviewing the open-ended responses for nonsensical responding and on the pre-registered exclusion criteria. Thus, we collected data from an additional 191 participants. In total, we collected data from 1039 individuals and excluded 288 participants from analyses. Participants were excluded if they provided a nonsensical response in response to an open-ended question (*n*=149), did not complete the study (*n*=45), incorrectly answered 2 of 3 comprehension questions regarding the prediabetes infographic (*n*=156), or if they incorrectly answered more than 1 of 3 attention checks (*n*=21). Participants were also excluded if the risk calculator website was down while they were taking the study (*n*=9), if they had missing data for the avoidance measures (*n*=42) or demographic variables (*n*=1), or if they responded inconsistently to two separate items asking if they learned their risk (*n*=22). There was a technological error that resulted in 16 participants not having data for the health self-efficacy, fatalism, or genetic determinism measures. Because one goal was to compare all of the health agency measures, we opted to exclude these 16 participants from all analyses. This resulted in a final analytic sample size of 735. Exclusion criteria were not mutually exclusive.

1. Behavioral Obligation Manipulation, Study 2

| High behavioral obligation condition | Low behavioral obligation condition |
| --- | --- |
| The American Diabetes Association has developed an accurate way to calculate your risk of prediabetes. You can learn your risk by answering several questions about yourself. If you score higher than a certain number, you are likely to have prediabetes and are at high risk of type 2 diabetes. **If you choose to calculate your risk today, and the calculator indicates you are high risk of prediabetes, you are recommended to make an appointment with a physician. The only way to get an official diagnosis of prediabetes is to get additional testing, including a blood test.** | The American Diabetes Association has developed an accurate way to calculate your risk of prediabetes. You can learn your risk by answering several questions about yourself. If you score higher than a certain number, you are likely to have prediabetes and are at high risk of type 2 diabetes. **If you choose to calculate your risk today, and the calculator indicates you are high risk of prediabetes, you will be given information about what to do next.** |
